# A data-driven model to describe and forecast the dynamics of COVID-19 transmission

**DOI:** 10.1371/journal.pone.0236386

**Published:** 2020-07-31

**Authors:** Henrique Mohallem Paiva, Rubens Junqueira Magalhães Afonso, Igor Luppi de Oliveira, Gabriele Fernandes Garcia

**Affiliations:** 1 Institute of Science and Technology (ICT), Federal University of São Paulo (UNIFESP), São José dos Campos, SP, Brazil; 2 Institute of Flight System Dynamics, Department of Aerospace and Geodesy, Technical University of Munich (TUM), Garching bei München, Bavaria, Germany; 3 Department of Electronics Engineering, Aeronautics Institute of Technology (ITA), São José dos Campos, SP, Brazil; University of Zambia, ZAMBIA

## Abstract

This paper proposes a dynamic model to describe and forecast the dynamics of the coronavirus disease COVID-19 transmission. The model is based on an approach previously used to describe the Middle East Respiratory Syndrome (MERS) epidemic. This methodology is used to describe the COVID-19 dynamics in six countries where the pandemic is widely spread, namely China, Italy, Spain, France, Germany, and the USA. For this purpose, data from the European Centre for Disease Prevention and Control (ECDC) are adopted. It is shown how the model can be used to forecast new infection cases and new deceased and how the uncertainties associated to this prediction can be quantified. This approach has the advantage of being relatively simple, grouping in few mathematical parameters the many conditions which affect the spreading of the disease. On the other hand, it requires previous data from the disease transmission in the country, being better suited for regions where the epidemic is not at a very early stage. With the estimated parameters at hand, one can use the model to predict the evolution of the disease, which in turn enables authorities to plan their actions. Moreover, one key advantage is the straightforward interpretation of these parameters and their influence over the evolution of the disease, which enables altering some of them, so that one can evaluate the effect of public policy, such as social distancing. The results presented for the selected countries confirm the accuracy to perform predictions.

## 1 Introduction

The geographic spread of a novel coronavirus (SARS-CoV-2) in Wuhan, China, in December 2019, characterized the emergence of a severe acute respiratory syndrome, afterward named COVID-19 [1–3]. Studies show that SARS-CoV-2 has a rapidly human-to-human and asymptomatic transmission, mainly by respiratory droplets, which makes it more contagious than Severe Acute Respiratory Syndrome (SARS) and Middle East Respiratory Syndrome (MERS), other well-known coronaviruses diseases, despite the contamination similarity. The new identified virus is closely related to SARS-CoV (79%) and MERS-CoV (50%) [1],[4]. Concerning the clinical aspects of COVID-19, the most common symptom reported was fever, followed by cough. A severe onset of the disease can lead to death due to alveolar damage and respiratory failure [1],[3],[5],[6].

Under these circumstances, COVID-19 has been spread through over 100 countries and all of the continents in a couple of months after its first confirmed case and it was declared, by the World Health Organization (WHO), a public health emergency of international matter in January 2020 [1],[7]. As of April 11th, 2020, about 1.6 million cases and 100.000 deaths were confirmed globally, showing an expressing breakthrough in Europe (839257 confirmed and 70565 deaths) and the region of the Americas (536664 confirmed and 19294 deaths) in comparison to the outbreak area (118549 confirmed and 4017 deaths). The most affected countries are the United States of America, Italy, Spain, China, Germany and France [8].

In the past two decades, there have been two coronavirus epidemics that also led to global health consternation, SARS in 2003 and MERS around 2012. It is noteworthy that COVID-19 has already killed more people than both of those diseases combined [9],[10]. Other epidemics have also ravaged the world and been considered an international emergency: H1N1 (2009), poliomyelitis (2014), Ebola (2014, 2019) and Zika (2016) [1].

Meanwhile, scientists have always had an essential role to play in the study of the dynamics of infectious diseases, particularly in mathematical modeling. The study of epidemiological mathematical models leads to deep understanding of the dynamics of epidemics, being an important tool to assess the potential effects of preventive and controlled measures, especially when their characteristics are still unclear [11],[12].

Recent works about COVID-19 models present different approaches to describe the proportions of its transmission and future numbers. The most common ones are the Susceptible-Infectious-Recovered/Death (SIRD) model [1],[13] and the Susceptible-Exposed-Infectious-Recovered (SEIR) model [14–16]; both derived from the Susceptible-Infectious-Recovered (SIR) pioneer model described by Kermack and McKendrick in 1927 [14–17]. Other works describe COVID-19 utilizing an exponential family model [18]; a second derivative model [19]; and Susceptible, Un-quarantined infected, Quarantined infected, Confirmed infected (SUQC) model [20].

These models are relevant to understanding the factors that alter the evolution of the disease. Furthermore, such models can be used to forecast evolution of the pandemic. In planning policies, a forecast of the number of infected and deceased individuals is of paramount importance. However, the models mentioned in the paragraph above do not account other classes, such as hospitalized individuals. The health infrastructure was placed under extreme pressure by the COVID-19 pandemic. Therefore, being able to forecast the number of hospitalized individual rises as an important task. Measures to mitigate the amount of simultaneously hospitalized individuals can be designed and evaluated to manage the facilities so that the deceased due to lack of proper treatment are minimized.

In the present paper, a SEIR model that includes the deceased and hospitalized is proposed to describe the dynamics of COVID-19. The availability of up-to-date data regarding the number of infected and deceased each day enables the estimation of the parameters so as to match the output of the model with the data from each country. Provided that, for each country two phases are assumed: (i) when no measures are taken such as reduction of social interaction and (ii) when public policies act to reduce the spread of the disease. Only the parameters linked to the transmissibility rates are allowed to change between these two phases. Moreover, the basic reproduction number is deduced for the proposed model and calculated from the parameters in the two phases.

One key feature of the proposed model is the straightforward connection between the parameters and their influence in the evolution of the disease. Therefore, the physical meaning of the parameters is clear, as opposed to black-box approaches and others that do not include the classes of individuals such as infected, recovered and so on, but rather focus on fitting curves. Therefore, it enables adjusting the parameters separately, each of which reflect real-world policy/behavior changes. For instance, transmissibility by infected individuals is a model parameter, which is affected by policy such as social distancing. This relationship allows one to perform simulations of different scenarios to predict the evolution of the disease under varied degrees of social distancing. This, in turn, is helpful to evaluate which policy is more promising. Moreover, the inclusion of the hospitalized class is another feature not present in the aforementioned models.

The remaining sections of this paper are divided as follows. The proposed model is presented in Section 2.1, whereas the basic transmission number is calculated as function of the model parameters in Section 2.2 and the parameter estimation problem is formally defined in Section 2.3. The results are presented in Section 3 and discussed in Section 4. Concluding remarks are given in Section 5.

## 2 Materials and methods

### 2.1 Model

Our proposed model is of the SEIR type and is inspired by the one used in [21] to successfully model the evolution of the Middle Eastern Respiratory Syndrome (MERS) coronavirus dynamic in the outbreak in South Korea in 2015. On the other hand, we introduce one extra class not present in [21], namely the class of deceased people, which play a relevant role in the evolution of the COVID-19. Therefore, the proposed model divides the population of interest in seven classes, as shown in Table 1. It is clear that each variable in Table 1 cannot assume negative values, as each represents the amount of individuals in a class.

**Table 1:** Classes of the proposed model.

Symbol	Meaning
*S* ≥ 0	Susceptible.
*E* ≥ 0	Exposed.
*I* ≥ 0	Infectious Symptomatic.
*A* ≥ 0	Asymptomatic.
*H* ≥ 0	Hospitalized.
*R* ≥ 0	Recovered.
*D* ≥ 0	Deceased.

The total population is represented by the symbol *N*. The introduction of the number of deceased is important in the case of COVID-19 due to two main effects: (i) in formulating policies, it is paramount to be able to predict the amount of deceased persons; (ii) these individuals are removed from the infected population and thus do not contribute to generate new infections. Albeit relevant in absolute terms and in proportion to the number of infected individuals, the deceased are proportionally low as compared to the total population, which justifies the adoption of Assumption 1 in our model.

**Assumption 1** *The population is deemed constant, i.e., N is not altered throughout the simulation of the model*.

Assumption 1 is reasonable, as the time interval for the simulation is short in terms of demographic changes and the amount of deceased people by the disease itself is not large enough to significantly alter the population of a country, as corroborated by the data.

Focus is placed on the relevant parameters to obtain a model that represents the main characteristics of the dynamics with minimal additional complexity. Following the development in [21], Assumption 2 deems zoonotic transmission not relevant for modeling purposes.

**Assumption 2** *Zoonotic transmission is not considered within the proposed model*.

Indeed, although it is suspected that the origin of the first cases is zoonotic, the evolution of the transmission has, since the first few hundred cases, in China been among humans only. In the other countries studied in the present work there has been no report of zoonotic transmissions.

Another relevant difference between our model and the one in [21] is the inclusion of an infection rate dependent on the asymptomatic individuals. It has been noticeable that many asymptomatic individuals transmit the virus SARS-CoV-2, therefore we introduced a term in the generation of new infections that reflects this fact. However, the infection rate of these individuals is not considered the same as that of infected symptomatic nor that of hospitalized ones, as we introduced a different coefficient for this rate in the generation of new infections.

**Assumption 3** *Deceased individuals came either from class H (hospitalized) or I (infected), but not from class A (asymptomatic)*.

Assumption 3 is made because our model parameters are estimated based on real data and the data available do not include the number of asymptomatic individuals that perish from the disease. The reported deceased come from the infected (*I*) or hospitalized (*H*) classes.

The resulting model under the aforementioned assumptions is given in (1), where we aimed at keeping the notation as close as possible to [21] to simplify comparison. The equations are presented in their general form; nevertheless, in order to represent Assumption 3, the value of *δ*_*A*_ is assumed equal to zero.

$$\begin{array}{c} \begin{array}{cc} \dot{S}= & -S\frac{\beta (I+\ell_{a}A+\ell H)}{N},\\ \dot{E}= & S\frac{\beta (I+\ell_{a}A+\ell H)}{N}-\kappa E,\\ \dot{I}= & \kappa \rho E-(\gamma_{a}+\gamma_{I}+\delta_{I})I,\\ \dot{A}= & \kappa (1-\rho )E-\mu A,\\ \dot{H}= & \gamma_{a}I-\left(\gamma_{r}+\delta_{H}\right)H,\\ \dot{R}= & \gamma_{I}I+\gamma_{r}H+\mu \left(1-\delta_{A}\right)A,\\ \dot{D}= & \delta_{H}H+\delta_{I}I+\mu \delta_{A}A. \end{array} \end{array}$$ (1)

**Fig 1:**
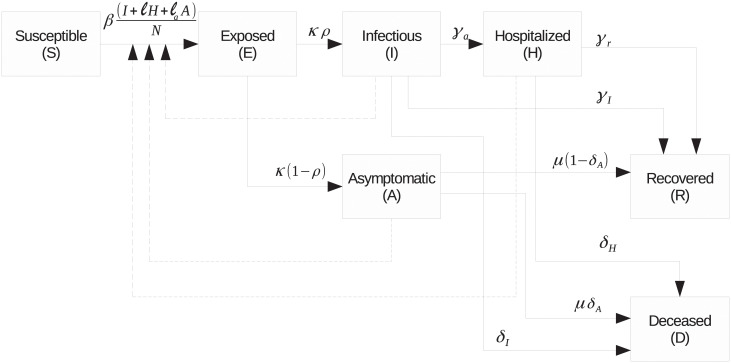
Block diagram representation of the model (1).

The meaning of the parameters in (1) is given in Table 2. A block diagram representation depicting the relationship between the variables in the model (1) is shown in Fig 1.

**Table 2:** Parameters of the proposed model.

Symbol	Meaning
*β* ≥ 0	human-to-human transmission rate per unit time (day)
*ℓ* ≥ 0	relative transmissibility of hospitalized patients
*ℓ*_*a*_ ≥ 0	relative transmissibility of asymptomatic infected
*κ* ≥ 0	rate at which an individual leaves the exposed class by becoming infectious (symptomatic or asymptomatic)
*ρ* ≥ 0	proportion of progression from exposed class *E* to symptomatic infected class *I*
*γ*_*a*_ ≥ 0	rate at which symptomatic individuals are hospitalized
*γ*_*I*_ ≥ 0	recovery rate without being hospitalized
*γ*_*r*_ ≥ 0	recovery rate of hospitalized patients
*μ* ≥ 0	rate of asymptomatic infectious that no longer transmit, becoming either recovered or deceased
*δ*_*A*_ ≥ 0	proportion of progression from asymptomatic class *A* to deceased class *D*
*δ*_*H*_ ≥ 0	death rate of hospitalized patients
*δ*_*I*_ ≥ 0	death rate of infected patients

### 2.2 Basic reproduction number

The basic reproduction number $R_{0}$ is the ratio of new infections from one single infected individual [22] in a totally susceptible population. It has theoretical value to understand how “infectious” a disease is, as larger $R_{0}$ indicates more spreading.

Introducing the state variable

$$\begin{array}{c} \text{x}=[\begin{array}{ccccccc} x_{1} & x_{2} & x_{3} & x_{4} & x_{5} & x_{6} & x_{7} \end{array}{]}^{⊤}=[\begin{array}{ccccccc} S & E & I & A & H & R & D \end{array}{]}^{⊤}, \end{array}$$ (2)

one may rewrite the system of ordinary differential equations (ODEs) (1) as

$$\begin{array}{c} \dot{\text{x}}=\text{f}\left(\text{x}\right). \end{array}$$ (3)

This nonlinear dynamic may have equilibrium points, i.e., points **x**_*eq*_ such that **f**(**x**_*eq*_) = **0**. Particularly interesting is the so-called disease-free equilibrium point in Definition 1.

**Definition 1** *A disease-free equilibrium point (DFE)* $\bar{\text{x}}$ *is an equilibrium point of the dynamic* (3) *such that* ${\bar{x}}_{1}=S=N$ *and* ${\bar{x}}_{i}=0$, *i* > 1.

It is easy to verify that (1) has a DFE.

Linearization of the model around the DFE is used in [23] to study the relationship between $R_{0}$ and stability. It has been demonstrated that if $R_{0}>1$, then the disease-free equilibrium point is unstable, i.e., an infected individual is enough to remove the system from the neighborhood of the DFE and infection of the population is possible. On the other hand, $R_{0}<1$ entails local asymptotic stability.

The authors argue in [23] that the definition of $R_{0}$ in terms of the model is not purely dependent on the model equations only. In fact, one must arbitrarily select which are the “infected” classes (state components of **x** in our notation). For our analysis we consider the infected states to be *x*_2_ = *E*, *x*_3_ = *I*, *x*_4_ = *A*, and *x*_5_ = *H*. The equations of interest for the analysis are the ODEs related to these state variables, namely

$$\begin{array}{c} {\dot{\text{x}}}_{I}=[\begin{array}{c} {\dot{x}}_{2}\\ {\dot{x}}_{3}\\ {\dot{x}}_{4}\\ {\dot{x}}_{5} \end{array}]=[\begin{array}{c} x_{1}\frac{\beta (x_{3}+\ell_{a}x_{4}+\ell x_{5})}{N}-\kappa x_{2}\\ \kappa \rho x_{2}-\left(\gamma_{a}+\gamma_{I}+\delta_{I}\right)x_{3}\\ \kappa \left(1-\rho \right)x_{2}-\mu x_{4}\\ \gamma_{a}x_{3}-\left(\gamma_{r}+\delta_{H}\right)x_{5} \end{array}]={\text{f}}_{I}\left(\text{x}\right), \end{array}$$ (4)

where ${\text{x}}_{I}=[\begin{array}{cccc} x_{2} & x_{3} & x_{4} & x_{5} \end{array}{]}^{⊤}$ are the infected states. One may now separate the dynamics **f**_*I*_(**x**) in two terms ${\text{f}}_{I}\left(\text{x}\right)=F_{I}\left(\text{x}\right)-V_{I}\left(\text{x}\right)$, where $F_{I}$ are the (positive) new infections and $V_{I}$ are the transitions between classes. From (4) one has

$$\begin{array}{c} F_{I}\left(\text{x}\right)=[\begin{array}{c} x_{1}\frac{\beta (x_{3}+\ell_{a}x_{4}+\ell x_{5})}{N}\\ 0\\ 0\\ 0 \end{array}]\;\text{and}\;V_{I}\left(\text{x}\right)=[\begin{array}{c} \kappa x_{2}\\ -\kappa \rho x_{2}+\left(\gamma_{a}+\gamma_{I}+\delta_{I}\right)x_{3}\\ -\kappa \left(1-\rho \right)x_{2}+\mu x_{4}\\ -\gamma_{a}x_{3}+\left(\gamma_{r}+\delta_{H}\right)x_{5} \end{array}]. \end{array}$$ (5)

Calculating the Jacobian of $F_{I}$ and $V_{I}$ with respect to **x**_*I*_ at $\bar{\text{x}}$ yields

$$\begin{array}{c} \frac{\partial F_{I}}{\partial {\text{x}}_{I}}\left(\bar{\text{x}}\right)=[\begin{array}{cccc} 0 & \beta & \beta \ell_{a} & \beta \ell \\ 0 & 0 & 0 & 0\\ 0 & 0 & 0 & 0\\ 0 & 0 & 0 & 0 \end{array}] \end{array}$$ (6)

and

$$\begin{array}{c} \frac{\partial V_{I}}{\partial {\text{x}}_{I}}\left(\bar{\text{x}}\right)=[\begin{array}{cccc} \kappa & 0 & 0 & 0\\ -\kappa \rho & \gamma_{a}+\gamma_{I}+\delta_{I} & 0 & 0\\ -\kappa (1-\rho ) & 0 & \mu & 0\\ 0 & -\gamma_{a} & 0 & \gamma_{r}+\delta_{H} \end{array}]. \end{array}$$ (7)

Assuming for the time being that the parameters are positive (an assumption that will be verified in the results in Section 3), $\frac{\partial V_{I}}{\partial {\text{x}}_{I}}\left(\bar{\text{x}}\right)$ is non-singular, thus we may calculate the inverse as

$$\begin{array}{c} {}[\frac{\partial V_{I}}{\partial {\text{x}}_{I}}\left(\bar{\text{x}}\right){]}^{-1}=[\begin{array}{cccc} \frac{1}{\kappa } & 0 & 0 & 0\\ \frac{\rho }{\gamma_{a}+\gamma_{I}+\delta_{I}} & \frac{1}{\gamma_{a}+\gamma_{I}+\delta_{I}} & 0 & 0\\ \frac{1-\rho }{\mu } & 0 & \frac{1}{\mu } & 0\\ \frac{\gamma_{a}\rho }{\left(\gamma_{r}+\delta_{H}\right)\left(\gamma_{a}+\gamma_{I}+\delta_{I}\right)} & \frac{\gamma_{a}}{\left(\gamma_{r}+\delta_{H}\right)\left(\gamma_{a}+\gamma_{I}+\delta_{I}\right)} & 0 & \frac{1}{\gamma_{r}+\delta_{H}} \end{array}]. \end{array}$$ (8)

The so-called next generation matrix is defined in [23] as $\frac{\partial F_{I}}{\partial {\text{x}}_{I}}\left(\bar{\text{x}}\right)[\frac{\partial V_{I}}{\partial {\text{x}}_{I}}\left(\bar{\text{x}}\right){]}^{-1}$ and $R_{0}$ is then determined as the spectral radius of the next generation matrix. From (6) and (8) one obtains

$$\begin{array}{c} \frac{\partial F_{I}}{\partial {\text{x}}_{I}}[\frac{\partial V_{I}}{\partial {\text{x}}_{I}}\left(\bar{\text{x}}\right){]}^{-1}=[\begin{array}{cccc} \beta (\frac{\rho }{\gamma_{a}+\gamma_{I}+\delta_{I}}+\ell_{a}\frac{1-\rho }{\mu }+\frac{\ell \gamma_{a}\rho }{\left(\gamma_{r}+\delta_{H}\right)\left(\gamma_{a}+\gamma_{I}+\delta_{I}\right)}) & \beta (\frac{1}{\gamma_{a}+\gamma_{I}+\delta_{I}}+\frac{\ell \gamma_{a}}{\left(\gamma_{r}+\delta_{H}\right)\left(\gamma_{a}+\gamma_{I}+\delta_{I}\right)}) & \frac{\beta \ell_{a}}{\mu } & \frac{\beta \ell }{\gamma_{r}+\delta_{H}}\\ 0 & 0 & 0 & 0\\ 0 & 0 & 0 & 0\\ 0 & 0 & 0 & 0 \end{array}]. \end{array}$$ (9)

The next generation matrix in (9) has only one positive eigenvalue and the remaining three are null. Therefore, the spectral radius of $\frac{\partial F_{I}}{\partial {\text{x}}_{I}}[\frac{\partial V_{I}}{\partial {\text{x}}_{I}}\left(\bar{\text{x}}\right){]}^{-1}$ is

$$\begin{array}{c} R_{0}=\beta (\frac{\rho }{\gamma_{a}+\gamma_{I}+\delta_{I}}+\ell_{a}\frac{1-\rho }{\mu }+\frac{\ell \gamma_{a}\rho }{\left(\gamma_{r}+\delta_{H}\right)\left(\gamma_{a}+\gamma_{I}+\delta_{I}\right)}). \end{array}$$ (10)

### 2.3 Parameter estimation

The parameters are estimated in order to match the total number of cases *C* and deceased *D* obtained from the model with the available data for each country, herein termed *C*_*real*_ and *D*_*real*_, respectively.

The cumulative number of infected individuals *C* at time *t* is obtained as follows:

$$\begin{array}{c} C\left(t\right)=\int_{0}^{t}\kappa \rho E(\tau )\;d\tau , \end{array}$$ (11)

where the integrand is the positive part of $\dot{I}$ in (1), which represents the number of new infected symptomatic individuals that enter class *I* per time unit.

As in the case of the MERS spread in South Korea reported in [21], some parameters of the model are allowed to assume two different values at two different periods, namely *β*, ℓ_*a*_ and ℓ, which are related to the rate of contagion of the population. These parameters can be affected by changes in policies by the authorities and reflect the control of the spread of the virus. Therefore, one has *β* = *β*(*t*), *ℓ*_*a*_ = *ℓ*_*a*_(*t*), and *ℓ* = *ℓ*(*t*) defined as:

$$\begin{array}{c} \begin{array}{cc} \beta (t)= & \left\{ \begin{array}{cc} \beta_{1}, & 0\leq t<T\\ \beta_{2}, & t\geq T \end{array}\right.\\ \ell_{a}\left(t\right)= & \left\{ \begin{array}{cc} \ell_{a,1}, & 0\leq t<T\\ \ell_{a,2}, & t\geq T \end{array}\right.\\ \ell (t)= & \left\{ \begin{array}{cc} \ell_{1}, & 0\leq t<T\\ \ell_{2}, & t\geq T \end{array}\right. \end{array} \end{array}$$ (12)

where *T* the is phase change time given in days and is also estimated. The data *C*_*real*_ and *D*_*real*_ are given as sequences *C*_*real*_(*i*) and *D*_*real*_(*i*) for $i=0,\;1,\;2,\;\ldots ,\;\bar{T}$, where 0 represents the day the number of infected people in the country reached 500 and $\bar{T}$ is the number of days of data used in the parameter estimation, which may vary for each country. All the remaining parameters are assumed constant.

The estimation is carried out via a constrained optimization problem enunciated as

**Problem 1**

$$\begin{array}{c} \text{p}*=\text{arg}\;\text{min}\sum_{i=0}^{\bar{T}}i\{[\frac{C\left(i\right)-C_{real}\left(i\right)}{C_{real}\left(\bar{T}\right)}{]}^{2}+[\frac{D\left(i\right)-D_{real}\left(i\right)}{D_{real}\left(\bar{T}\right)}{]}^{2}\} \end{array}$$ (13)

*subject to*

$$\begin{array}{cc} \text{p} & =[\begin{array}{cccccccccccccccc} T & \beta_{1} & \beta_{2} & \ell_{1} & \ell_{2} & \ell_{a,1} & \ell_{a,2} & \kappa & \rho & \gamma_{a} & \gamma_{I} & \gamma_{r} & \mu & \delta_{H} & \delta_{I} & I(0) \end{array}{]}^{⊤}, \end{array}$$ (14)

p≥0. (15)

In (15) the inequality in considered element-wise. The result p* is the vector of optimal parameter values, i.e., values that minimize (13) while satisfying the constraints (14) and (15). This optimization problem is solved using a Sequential Quadratic Program algorithm [24], adopting as starting point the parameters used in [21] to describe the MERS epidemic.

### 2.4 Data source

The COVID-19 data used in this paper, with the number of infected and deceased people in each country, were downloaded from the website of the European Centre for Disease Prevention and Control (ECDC) [25] on June 19th, 2020.

## 3 Results

The resulting parameters from the estimation are shown in Table 3, where the numbers in subscript “1” and “2” indicate whether the parameter refers to the first or second period, respectively. The basic reproduction number was also calculated for both periods and is depicted separately in Table 3, since it is not a model parameter.

**Table 3:** Results of the parameter estimation for the selected countries.

Parameter	Country					
	China	Italy	Spain	France	Germany	USA
T (days)	18	30	23	26	24	51
*β*_1_	0.334	0.189	0.382	0.298	0.135	0.303
*β*_2_	0.140	0.081	0.160	0.129	0.055	0.130
*ℓ*_1_	0.673	8.000	7.690	8.000	4.800	0.851
*ℓ*_2_	0.135	8.000	6.490	8.000	1.130	0.851
*ℓ*_*a*.1_	8.000	0.649	3.900	8.000	4.900	4.090
*ℓ*_*a*.2_	8.000	0.649	3.900	8.000	4.900	0.820
*κ*	0.440	0.284	0.362	0.309	0.578	1.330
*ρ*	0.053	0.270	0.102	0.033	0.021	1.010
*γ*_*a*_	0.503	0.224	0.116	0.300	0.542	0.055
*γ*_*I*_	0.263	0.040	0.063	0.020	0.050	0.296
*γ*_*r*_	0.141	0.240	0.281	0.131	0.036	0.018
*μ*	1.640	0.146	1.030	1.530	0.302	0.828
*δ*_*H*_	0.008	0.023	0.019	0.029	0.003	0.00029
*δ*_*I*_	0.003	0.023	0.016	0.018	0.002	0.023
$R_{0.1}$	1.62	2.00	2.09	1.98	2.47	2.90
$R_{0.2}$	0.66	0.86	0.84	0.85	0.91	1.26

**Fig 2:**
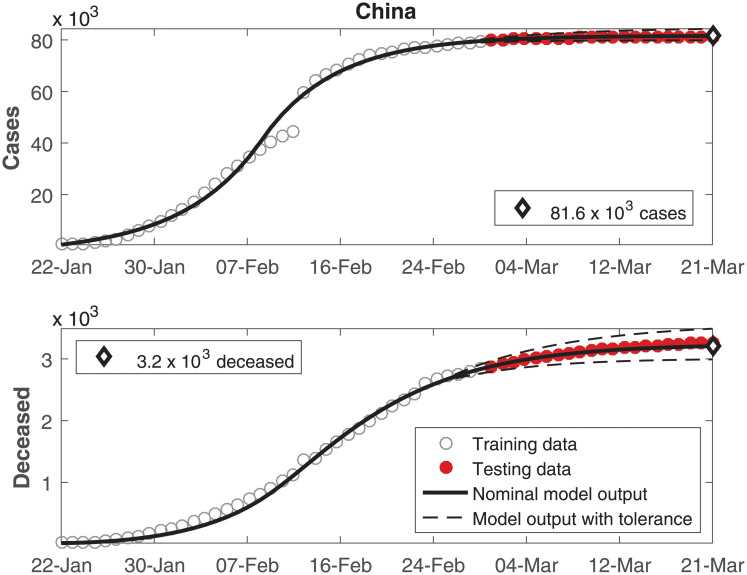
Cumulative number of infected individuals *C* and number of deceased individuals *D* for China.

**Fig 3:**
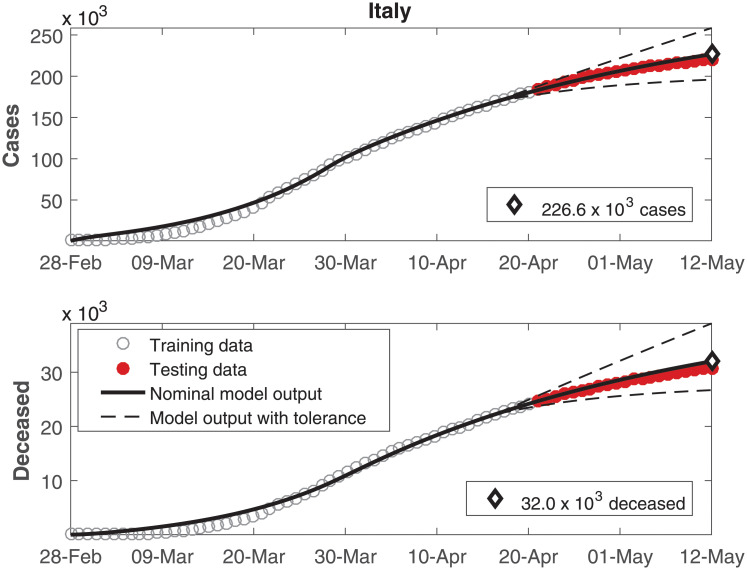
Cumulative number of infected individuals *C* and number of deceased individuals *D* for Italy.

**Fig 4:**
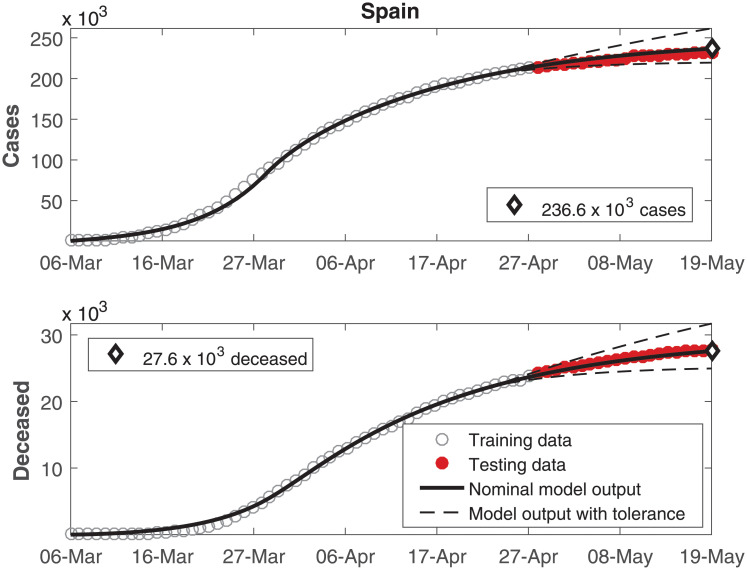
Cumulative number of infected individuals *C* and number of deceased individuals *D* for Spain.

**Fig 5:**
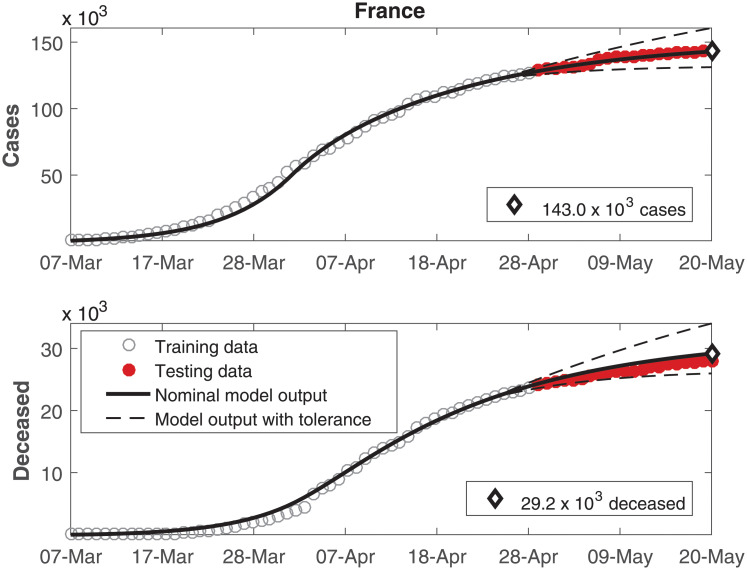
Cumulative number of infected individuals *C* and number of deceased individuals *D* for France.

**Fig 6:**
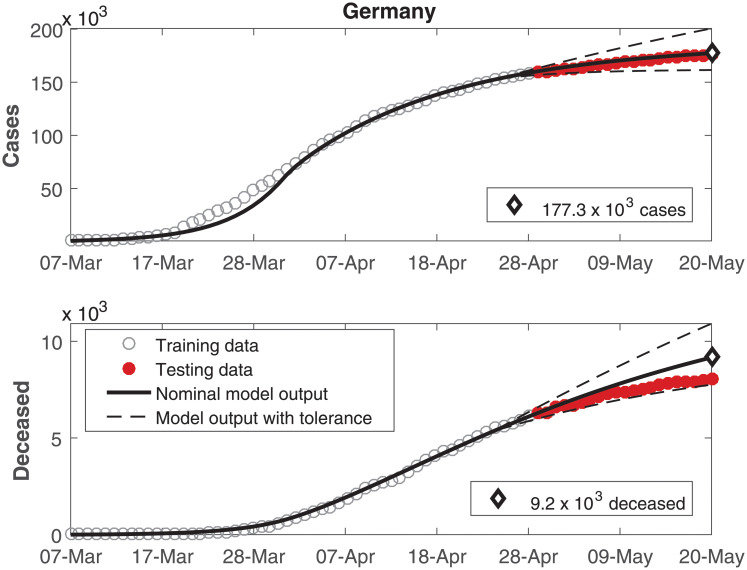
Cumulative number of infected individuals *C* and number of deceased individuals *D* for Germany.

**Fig 7:**
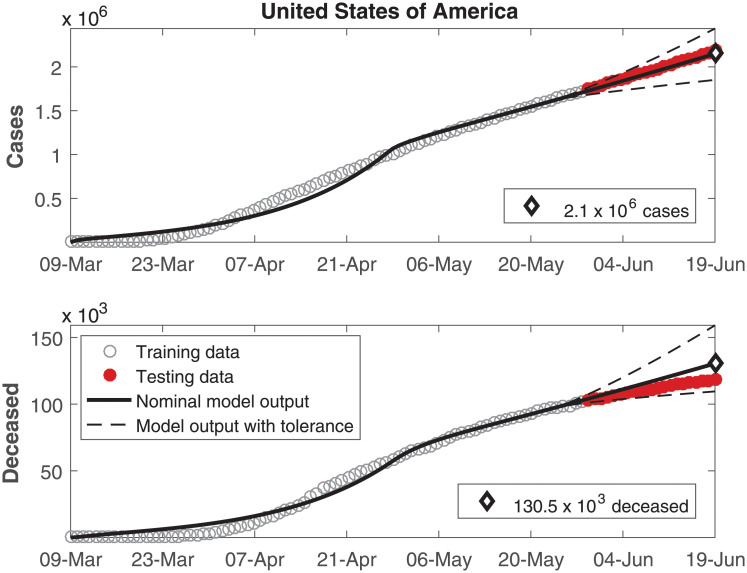
Cumulative number of infected individuals *C* and number of deceased individuals *D* for the United States of America.

The data of number of cumulative infected individuals and deceased per day as well as the model output for these variables are shown in Figs 2, 3, 4, 5, 6 and 7, for China, Italy, Spain, France, Germany, and the USA, respectively.

In these figures, data from the last 21 days are used as testing data, in order to allow a comparison between the model predictions and the real outcome of the disease. In these cases, the black continuous lines represent the predictions with the nominal parameters from Table 3 and the dashed lines are variations when selected parameters are changed by ±30%.

## 4 Discussion

In Figs 2 to 7, it can be seen that there is a good match between the model output and the observed data. Furthermore, the model presents accurate forecasts of the disease progression. There are some variations between the nominal prediction and the observed data, but within the established tolerances. The uncertainty in the prediction increases as more time has passed since the date when the model was calibrated; these uncertainties are reduced if the model parameters are updated periodically.

Although an overall good match was obtained for China (Fig 2), a mismatch can be seen on the four days before 12-feb. This difference is ascribed to the fact that the country changed the notification methodology on 12-feb [26], reporting more than 15000 cases on a single day, which caused a discontinuity in the cumulative cases on this date. The model cannot reproduce such discontinuity, for it was not designed to consider changes in the nature of observed data.

In general, the parameters reported in Table 3 are consistent (in terms of magnitude order) with the ones described previously in [21] for the MERS outbreak in South Korea. It is important to remark that the values of the parameters are expected to be different among the various countries, as the contagion, hospitalization and death rates are affected by social habits, demographic conditions, governmental action, quality and coverage of the health systems, etc.

The value of $R_{0}$ is of particular interest. It is expected that $R_{0}>1$ in the first phase during the spread and $R_{0}<1$ after the authorities take measures to control the spread. A reduction in *ℓ*_*a*_ combined with a reduction in *β* shows where the focus to reduce contagion is most effective: reducing social contact at all cost so that both symptomatic but also importantly asymptomatic individuals stop transmitting the virus.

The estimated parameters are specially trustworthy for countries that already reached the asymptotic behavior of the curves. For countries where the epidemic is still ongoing, it is expected that the parameter estimates are more sensitive to new data.

According to our estimations, five countries have reached a second phase with $R_{0}<1$, meaning that the epidemic is under control and converging to an equilibrium. On the other hand, the USA have also reached a second stage with a lower $R_{0}$, but still higher than 1, indicating that the number of infections is decelerating but is not under control yet.

A *δ*_*I*_ value higher than *δ*_*H*_ was to be expected, indicating a lower rate of mortality in hospitalized patients. However, that was observed only in the USA. In the other countries in this study, the value of *δ*_*H*_ is higher or very similar to the value of *δ*_*I*_. In these cases, this behavior might be justified by the most critical cases being treated in the hospitals, therefore being associated to a higher mortality rate. It is interesting to remark that the predictions of the number of deceased in Germany in Fig 6 and the USA in Fig 7 presented a larger mismatch both as compared to the other countries as well as compared to the number of cases within Germany and the USA themselves. Just as social distancing, more careful hygiene and other such measures can reduce the transmissibility, it might be expected that the development of protocols for treatments using the knowledge developed by medical experts might reduce the death ratios beyond model predictions. In this case, the parameters related to the deceases might demand another phase for estimation.

The relatively high values of *ℓ* and *ℓ*_*a*_ indicate that the contagion rate is higher with hospitalized patients and asymptomatic individuals. The higher transmissibility in the hospitals was to be expected, whereas the higher contagion rate of asymptomatic individuals may be ascribed to them not taking the same precautions as symptomatic people to avoid spreading the disease. The value of *β* should not be analyzed alone, for it provides a better insight when combined with the values of *ℓ* and *ℓ*_*a*_.

In all countries, it can be seen that *β* indeed decreased in the second phase. However, for some cases *ℓ*_*a*_ remained constant. One conjecture for such results lies in the lack of data for estimating the parameters: the used database from ECDC does not include asymptomatic individuals. In some countries where testing on a larger scale took place, one could try to use such data to more accurately estimate the parameters associated with the asymptomatic class. However, this data is bound to be less trustworthy that those for infected symptomatic individuals and deceased, for which the data are collected daily and one can say that the reports are very close to the reality. On the other hand, the asymptomatic will remain an estimate as long as a representative part of the population is not tested. Moreover, this would have to be repeated at regular intervals. Such repeated large scale testing is impractical in reality, therefore the lack of data covering asymptomatic infected individuals with the same regularity and within tight confidence intervals as those for symptomatic and deceased.

Reducing the values of the parameters *β*, *ℓ*_*a*_, and ℓ are the focus of the authorities in controlling the spread, therefore allowing them to change throughout the estimation is a means to address changes promoted by governmental action. This is a point where the proposed model and estimation are useful for prediction. The model enables not only prediction with the current estimates of the parameters, but also simulations with changes in these parameters. This permits decision makers to assess the impact of changes in terms of the rate of social contact avoidance that is necessary to enforce in a country as means to control the spread. Lately, many ways to measure the social contact reduction have been employed, such as using data from movements of cell phones.

## 5 Conclusion

This paper proposed a SEIR model to describe the COVID-19 epidemics. Its parameters were estimated with a numerical optimization algorithm, based on data from the European Centre for Disease Prevention and Control. An analysis was presented for the six countries where the pandemic is widely spread: China, Italy, Spain, France, Germany, and the USA. A good match between theoretical and observed data was achieved and a forecast was presented.

Since the model is data-driven, one drawback is that it cannot be used for countries where the epidemic is at a very early stage. Nevertheless, in the lack of local data, the pandemic behavior can still be estimated using parameters from another country. However, in this case, the uncertainty in the estimations would be higher.

It should be pointed out that forecasting the future is always imprecise, and that predictions are better for the near future. The outcome of the epidemic might be significantly altered by changes in governmental policy, such as enforcing or releasing measures to reduce social contact, or by other factors such as overload of the health care systems. Therefore, the model parameters should be updated as soon as new data become available. Although this paper presented a three-week ahead forecast, the authors recommend to update such parameters at least on a weekly basis.

Reducing the basic reproduction number $R_{0}$ below one is known to represent that the spread is under control. Our developed formula for $R_{0}$ shows a strong influence on the human-to-human transmission rate *β*, followed by the relative transmissibility of hospitalized patients *ℓ* and of the asymptomatic infected *ℓ*_*a*_. By evaluating the estimated values authorities can grasp which actions are more likely to yield more meaningful results. For example, considering that for a country the value of *β* has stalled, whereas *ℓ*_*a*_ is still relatively high, measures to reduce the social contact of asymptomatic individuals appear as promising alternatives, instead of simply isolating the symptomatic individuals.

The forecasts are useful for planning purposes. For instance, when authorities must decide whether to invest in augmenting the capacity of hospitals in the coming weeks, it is of paramount importance to have a precise forecast of the number of hospitalized individuals. Besides enabling that, our proposed model involves parameters that have straightforward meaning related to the spread of the disease. Therefore, not only a nominal case can be considered, but the parameters may be varied within reasonable bounds under the scrutiny of specialists to yield worst and best case predictions, enhancing the awareness level of authorities in the process of decision making.

Future work includes defining a fixed target date and value for the maximal number of cumulative infected individuals and then optimize the parameters *β*, *ℓ*_*a*_, and *ℓ* so as to determine whether authorities should aim at more or less stringent social contact control measures, for instance. Another opportunity for future enhancement would be to allow for variations in *δ*_*H*_ to model the overload of the hospitals. This, however, requires available data of the number of hospitalized individuals and available infrastructure.
